# Paper-Based Sensing Device for Electrochemical Detection of Oxidative Stress Biomarker 8-Hydroxy-2′-deoxyguanosine (8-OHdG) in Point-of-Care

**DOI:** 10.1038/s41598-017-14878-9

**Published:** 2017-11-06

**Authors:** Gabriela V. Martins, Ana P. M. Tavares, Elvira Fortunato, M. Goreti F. Sales

**Affiliations:** 1 BioMark/CINTESIS-ISEP, School of Engineering of the Polytechnique School of Porto, 4200-072 Porto, Portugal; 2 i3N/CENIMAT, Department of Materials Science, Faculty of Sciences and Technology, Universidade NOVA de Lisboa and CEMOP/UNINOVA, Campus de Caparica, 2829-516 Caparica, Portugal

## Abstract

This work presents a cost-effective, label-free in point-of-care (POC) biosensor for the sensitive detection of 8-hydroxy-2′-deoxyguanosine (8-OHdG), the most abundant oxidative product of DNA, that may allow a premature assessment of cancer disease, thereby improving diagnosis, prognostics and survival rates. The device targets the direct detection of 8-OHdG by using for the first time a carbon-ink 3-electrode on a paper substrate coupled to Differential Pulse Voltammetry readings. This design was optimized by adding nanostructured carbon materials to the ink and the conducting polymer PEDOT, enhancing the electrocatalytic properties of the sensor towards 8-OHdG detection. Meanwhile, the ability of this oxidative stress biomarker to undertake an oxidation reaction enabled the development of the sensing electrochemical device without the need of chemical probes and long incubation periods. This paper-modified sensor presented high electrochemical performance on the oxidation of 8-OHdG with a wide linear range (50–1000 ng/ml) and a low detection limit (14.4 ng/ml). Thus, our results showed the development of a direct and facile sensor with good reproducibility, stability, sensitivity and more importantly, selectivity. The proposed carbon-based electrochemical sensor is a potential candidate to be miniaturized to small portable size, which make it applicable for *in-situ* 8-OHdG sensing in real biological samples.

## Introduction

Oxidative stress (OS) constitutes an important imbalance established between reactive oxygen species (ROS) and the oxidant system defence in biological organisms^1^. As a consequence, the radical species that are formed interact directly with the biomolecules present in the cells, such as, proteins, phospholipids and nucleic acids, causing cell degeneration and death^2,3^. In parallel, specific molecules are produced and their quantification can be used as OS biomarkers for different biological matrices^4^. For instance, recent studies have shown that high concentrations of these biomarkers can be associated with degenerative diseases, such as Alzheimer, hypertension, type II diabetes and several types of cancer^5,6^. Furthermore, the physiological level of these biomarkers in biological tissues or fluids are also influenced by other conditions, as age^7^ and gender^8^, for instance.

Overall, OS biomarkers include compounds derived from nucleic acid oxidation, such as, 8-hydroxy-2′-deoxyguanosine (8-OHdG), which has been directly linked to increased risk of developing several degenerative and cancer diseases^9^. Among others, its known mutagenicity is one of the major reasons for recognizing 8-OHdG as the most frequently used biomarker of oxidative DNA damage^10^. Although the detection of oxidative damage is being widely applied in human research and clinical applications, we still need more data about the main factors that determine the basal levels of these biomarkers among general population. Recently, some interesting studies have suggested that some variables, such as, age, sex, alcohol consumption, level of education, the time season of sample collection and exposure to heavy metals are implicated with 8-OHdG quantification^11,12^.

The relevance of 8-OHdG as OS biomarker is confirmed by the numerous methods published in the literature aiming its determination. Gutiérrez *et al*. reported self-assembled monolayers on gold-modified electrodes with dendrimers for 8-OHdG detection with a limit of detection of 1.2 nM^13^. An electrochemiluminescence immunosensor based on platinum (Pt) electrode modified with carbon quantum dots and Au/SiO_2_ core-shell nanoparticles was designed for a rapid and selective detection of 8-OHdG for biological samples^14^. A sensitive automated flow immunosensor has been developed for detection of urinary 8-OHdG at concentrations of 0.05 ng/ml^15^. Also, the combination of sensitive semi-conducting silicon nanowire with the specificity of immunoassays have resulted in a biosensor device to detect few nanomolar concentrations of 8-OHdG^16^. Recently, we have also presented an electrochemical biosensor using a molecular imprinting approach to monitor urinary 8-OHdG down to the pmol/L level^17^.

Other works used electrochemical sensing approaches. Some of these rely on the electro-activity of DNA bases, as a way to measure its biomolecular damage^18–20^. In this context, guanine and adenine were immobilized as DNA bases on carbon electrodes and their direct electrochemical response measured, as an indirect measure of DNA damage detection^21,22^. Other approaches aim the direct determination of OS biomarkers. In these, carbon-based electrodes were widely applied, mostly due to their physical and electronic characteristics^23^. Li *et al*. performed some electrochemical studies by using conducting polymer poly(3-methylthiophene) (P3MT) modified glassy carbon electrodes for an electrochemical detection of urinary 8-OHdG, enabling a limit of detection of 0.10 μM^24^. Langmaier *et al*. have investigated the oxidation reaction of 8-oxo-2′-deoxyguanosine on glassy carbon (GC), Pt, gold (Au) and SnO_2_ electrodes^25^. It was observed that the rate of charge transfer reaction depends on the nature of the electrode material, following the sequence GC > Pt, Au >> SnO_2_. More interestingly, these effects can be related to the density of the active surface sites (GC) or to the degree of oxidation of the electrode surface (Pt, Au, SnO2). In sum, an overview of the functionalization of new biosensors showed that in addition to the improvement of the sensing element, the importance of the support material is also crucial.

Overall, electrochemical sensors are increasingly gaining attention due to their high sensitivity (low detection limits), small dimensions, low cost, easy automation and operation, as can be seen on Table 1. Special importance has been given to carbon electrodes and the use of nanostructured materials, such as graphene, nanoparticles and carbon nanotubes. These nanomaterials are currently used as a surface modification approach to accelerate electron transfer and enhance the electrochemical activity of biomolecules due to their intrinsic characteristics such as, higher surface area, good conductivity and signal stability^26–28^. Even after such amazing developments, an urging need for OS assessment in point-of-care (POC) remains. Specifically, there is a gap regarding rapid, portable, inexpensive and simple screening platforms for biomarker analysis.

**Table 1:** Comparison of different electrochemical sensors for determination of 8-OHdG.

Working electrode/Substrate	Working electrode/Modification	Detection technique	Range of linear concentration dependence	Type of Sensor	Real application	Reference
2 electrode system/CNT conductive paper	—	Chrono-amperometric (*and colorimetric*)	0–530 nM	*Immuno-sensor*	Urine samples	37
3 electrode system/GCE electrode	SWCNTs- Nafion dispersion	DPV	0.03–1.25 mM		Urine samples	40
			1.25–8.75 mM			
3 electrode system/GCE electrode	Graphene- Nafion film	LSV	0.07–3.64 mM		Urine samples	39
			3.64–16.24 mM			
			16.24–33.04 mM			
3 electrode system/GCE electrode	MWCNT film	LSV	80–5000 nM		Urine samples	60
3 electrode system/GCE electrode	Poly(3-methyl thiophene)	CV	0.700–35.0 mM		Urine samples	24
			35.0–70.0mM			
3 electrode system/GCE electrode	Poly(indole-5-carboxylic acid) and chitosan	DPV	0.35–35305 nM	*Immuno-sensor*	Urine samples	59
3 electrode system/Pt electrode	Carbon quantum dot coated with Au/SiO_2_ core-shell nanoparticles	ECL	0.71–706 nM	*Immuno-sensor*	Milk samples	14
3 electrode system/Au electrode	Phenol polymer	EIS	0.35–353 pM	*MIP*	Urine samples	17
3 electrode system/GCE electrode	Sulfur-doped graphene	DPV	0.002–20 mM		Urine samples	41
3 electrode system/GCE electrode	DNA functionalized graphene nanosheets	CV	0.0056–1.155 mM		Urine samples	38
			1.155–11.655 mM			
			11.655–36.155 mM			

Au: gold; CNT: carbon nanotube; CV: cyclic voltammetry; DPV: differential pulse voltammetry; ECL: electrochemiluminescence; EIS: electrochemical impedance spectroscopy; GCE: glass carbon electrode; HPLC-ECD: High Performance Liquid Chromatography with Electrochemical Detection; LSV: linear sweep voltammetry; MIP: molecularly imprinting polymer; MWCNT: multi walled carbon nanotubes; PAMAM: poly(amidoamine); Pt: platinum; SAM: self-assembled monolayers; SWCNTs: single walled carbon nanotubes.

In turn, paper-based sensors have become a promising platform for *lab-on-a-chip* devices, offering high selectivity and sensitivity for application areas such as, health diagnostic, food quality control and environmental monitoring^29^. In particular, a recent overview about diagnostic paper-based biosensors has discussed the integration of nanomaterials for the detection of nucleic acids, proteins and cells^30^. Due to their simplicity, portability and low cost, the most commonly used analysis techniques applied on paper-based sensing devices are colorimetric^31^, electrochemical^32,33^, chemiluminescent^34^ and electrochemiluminescent^35,36^. In addition, these paper-based biosensors are also aiming to become an environmentally safe alternative to the conventional Screen Printed Electrodes (SPE) for screening OS biomarkers. Moreover, in some cases, the reproducibility and sensitivity characteristics of the electrodes are poor and/or the fabrication process is complex, thereby hindering scale-up processes and POC use. Although there are already very few studies performed on paper electrode devices for quantitative analysis of 8-OHdG biomarker, they still require the immobilization of antibodies^37^.

**Figure 1:**
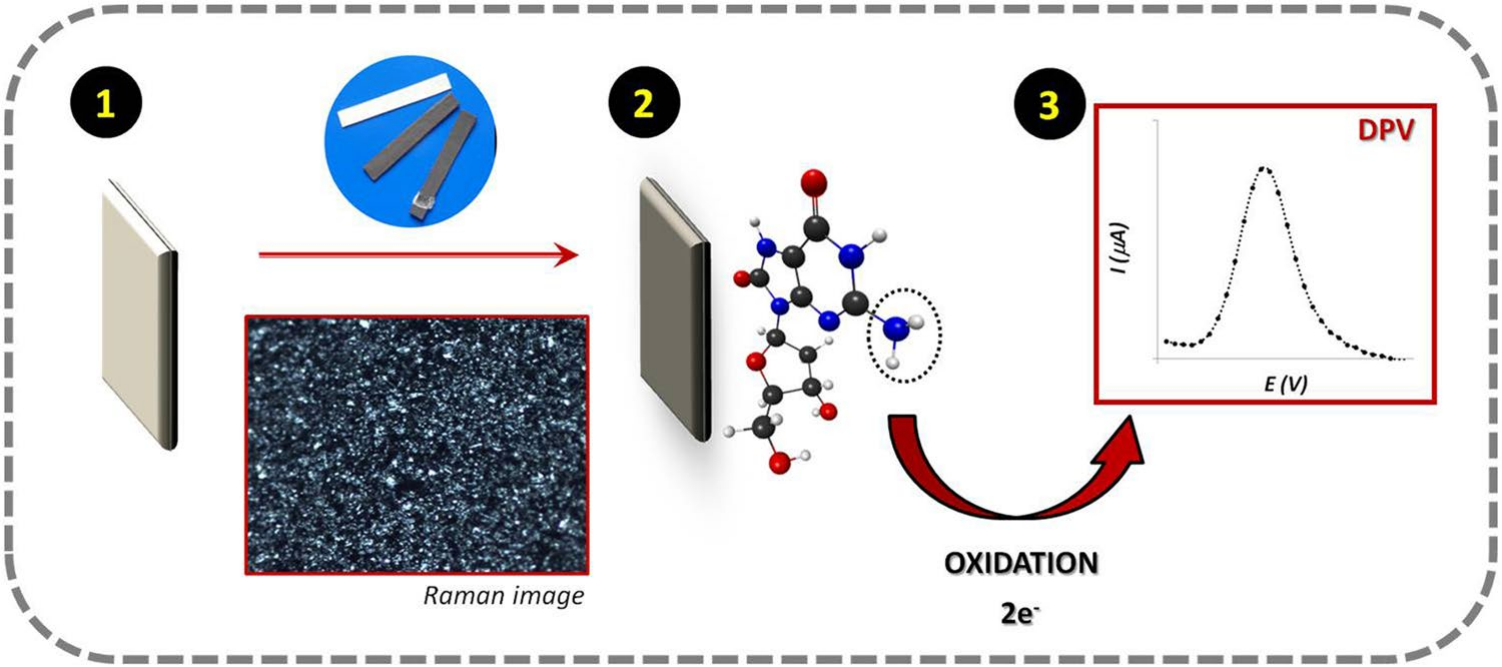
Schematic representation of the oxidation process of 8-OHdG molecule followed on a conductive carbon paper substrate: (1) hydrophobic white paper as substrate; (2) conductive carbon-coated paper; (3) *in-situ* electrochemical measurement.

Thus, this work aims the development of low cost *easy-to-use* label-free paper-based, electrochemical biosensor for the determination of OS biomarkers, which targets 8-OHdG as proof-of-concept. For this, we have investigated the redox behaviour of 8-OHdG on different carbon-modified surfaces and the optimization conditions for its selective detection in biological samples. The redox reaction of 8-OHdG is presented elsewhere^25^ and the detection process of the modified electrodes can be found in Fig. 1. Here, the electrochemical performance of the biomarker 8-OHdG at the modified-paper electrode was followed by means of differential pulse voltammetry (DPV), and the several electrochemical and chemical variables optimized and evaluated.

## Results and Discussion

### Electrochemical behaviour of 8-OHdG

**Figure 2:**
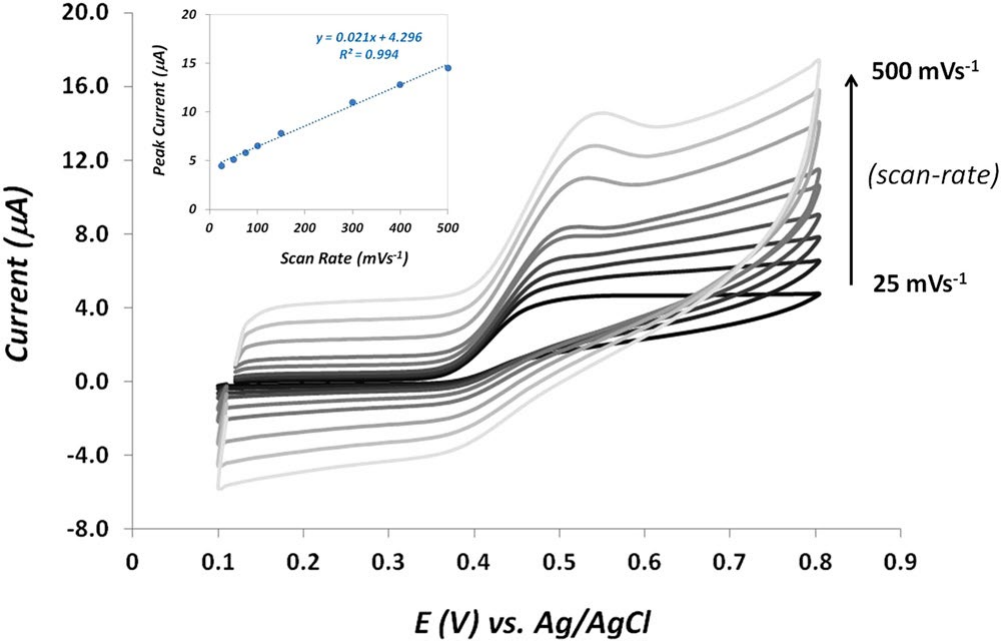
Successive cyclic voltammograms performed in PBS at pH 7.4 with 8-OHdG molecule at different scan rates. Inset: calibration plot of the 8-OHdG oxidation peak current versus scan rate.

Figure 2 shows the cyclic voltammograms of 8-OHdG on PBS pH 7.4 performed for different scan rates onto the surface of the paper-modified electrodes. Here, a well-defined oxidation peak appeared around +0.42 V and a small reduction peak was visible at +0.40 V. Thus, the peak shape of the CV demonstrated a typical quasi-reversible electrochemical reaction. In agreement with previous electrochemical studies, it was also observed that the oxidation peak potential shifted gradually towards more positive values with the increase of scan-rate^24^.

Additionally, the influence of scan-rates on the electrochemical oxidation of 8-OHdG on these graphite-coated electrodes was also investigated. As can be seen on the inset figure, a linear relationship (R^2^ = 0.994) between the anodic peak current and the scan rate is evident, suggesting that the electrochemical oxidation process of 8-OHdG is mainly adsorption-controlled. Our results are in good agreement with other studies performed on various carbon-modified surfaces^38,39^. Herein, CV was employed to determine the oxidation potential of 8-OHdG to be used in further electrochemical experiments (+0.41 V), a value that is in agreement with other similar electrochemical sensors^37,40,41^.

### DPV analysis of 8-OHdG on paper-modified electrodes

Graphitic materials are known for their good structural, electronic, mechanical, optical, thermal and chemical properties^42^. In the last years, carbon-based nanomaterials, such as, graphene^43^, nanotubes^44^, nanoparticles^45^ and conductive polymers^46^ are being widely employed for biosensing devices. Due to their high surface area and high electrical conductivity, these electrochemical sensors have become a potential alternative to the conventional labour intensive and time consuming assays that are currently used for biomarker analysis. Since the electrochemical performance of the sensor is mainly affected by coating characteristics, developing a suitable ink for a specific target should be an important issue to design novel sensing devices.

**Figure 3:**
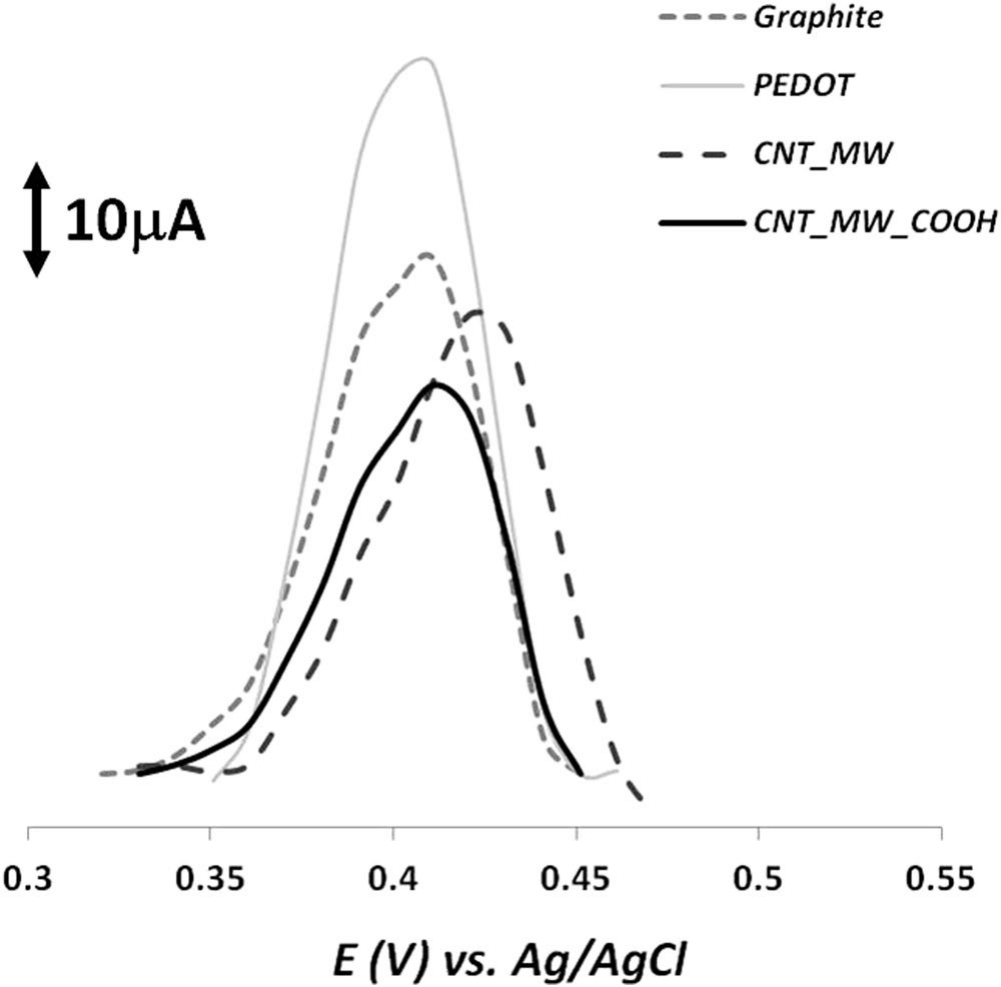
DPV detection of 200 ng/ml 8-OHdG solution in PBS pH 7.4 on different graphite-based electrodes prepared after the incorporation of various nanomaterials dispersed in the graphite ink, such as, PEDOT nanoparticles, CNTMW and CNTMW-COOH.

Under the scope of this work, differential pulse voltammetry (DPV) was chosen as a highly sensitive and selective electrochemical tool for quantitative analysis, with great application for nucleic acid sensing^22,47^. Meanwhile, the ability of 8-OHdG to undertake an electrochemical oxidation through 2-electron transfer reaction on carbon surfaces has already been reported^48^. So, in order to enhance the DPV signal of the oxidation of 8-OHdG on the paper-modified electrodes, we have incorporated and tested the effect of some highly conductive materials into the graphite-ink. Figure 3 displays the corresponding peaks of oxidation of 8-OHdG on different graphite-based electrodes, prepared through the incorporation of some conductive materials such as, poly(3,4-ethylenedioxythiophene) (PEDOT) nanoparticles, carbon nanotubes multi-walled (CNTMW) and carboxylated carbon nanotubes multi-walled (CNTMW-COOH). Our data showed that the presence of the conducting polymer PEDOT seems to greatly improve the electro-catalytic properties of the substrate leading to an enhancement of the electrochemical response of 8-OHdG in comparison with the electrode coated with the graphite alone. This outcome can be attributed to the symbiotic combination of the good electronic mobility of the PEDOT polymer with the high surface area of the nanoparticles resulting in a facilitated charge transfer for 8-OHdG redox biomarker. Although nano-based materials are often employed in order to accelerate electron transference, here the introduction of nanotubes during the ink production did not reflect on a improvement of the peak current intensity. Hence, the obtained oxidation potentials were quite similar between the different carbon-based surfaces and so, the graphite ink doped with PEDOT was chosen for the further experiments.

### Characterization of the paper-modified electrodes

Raman spectroscopy has become a popular, powerful and non-invasive tool to characterize the structural organization of carbon and related materials. Figure S1 shows the RAMAN spectra for the different graphite-based electrodes prepared with the inks doped with various nanomaterials. Firstly, in all samples, the typical G and D bands appeared at 1581 cm^−1^ and 1350 cm^−1^, respectively, which is in accordance with previous Raman spectrum for graphite samples^49–51^. The G band is often associated to the stretching of the C-C in graphitic materials, common to all sp^2^ carbon systems, and the D band is assigned to the presence of disorder in the sp^2^-hybridized carbon system^52^. In addition, another peak was also clearly visible around 2700 cm^−1^ that is assigned to the 2D band. As expected, the spectra of all observed materials are quite similar because the main constituent of the doped inks was graphite. Nevertheless, by analysing the intensity peak ratio between the D and G bands (I_D_/I_G_), we are able to evaluate the level of disorder or defects within the carbon material. The most common information extracted from the direct comparison between Raman spectrum is that an increasing tendency of the I_D_/I_G_ intensity ratios reflects the presence of additional structural disorder. Here, the I_D_/I_G_ ratios of the graphite ink alone, with PEDOT, MWCNT and COOH-MWCNT were 0.30, 0.17, 0.40 and 0.84, respectively. An interesting work have demonstrated a correlation between the number of edge plane type defects on graphite-based substrates and the voltammetry of guanine oxidation. The authors have concluded that as the number of defects sites on the electrode surface increases, there is a small shift in peak potential and, more importantly, the peak height is enhanced^53^. In our case, it was interesting to observe that the carbon-based material with less level of disorder (less ratio I_D_/I_G_, 0.17) was achieved for the ink doped with PEDOT nanoparticles, which enabled the higher intensity current for DPV signaling of 8-OHdG oxidation. This is consistent with the fact that PEDOT displays a structure where carbon atoms hold an sp^2^ hybridization. In addition, the material assigned with the higher number of structural defects (high ratio I_D_/I_G_, 0.84) was found to give the weaker DPV response.

### Optimization of DPV experimental conditions

**Figure 4:**
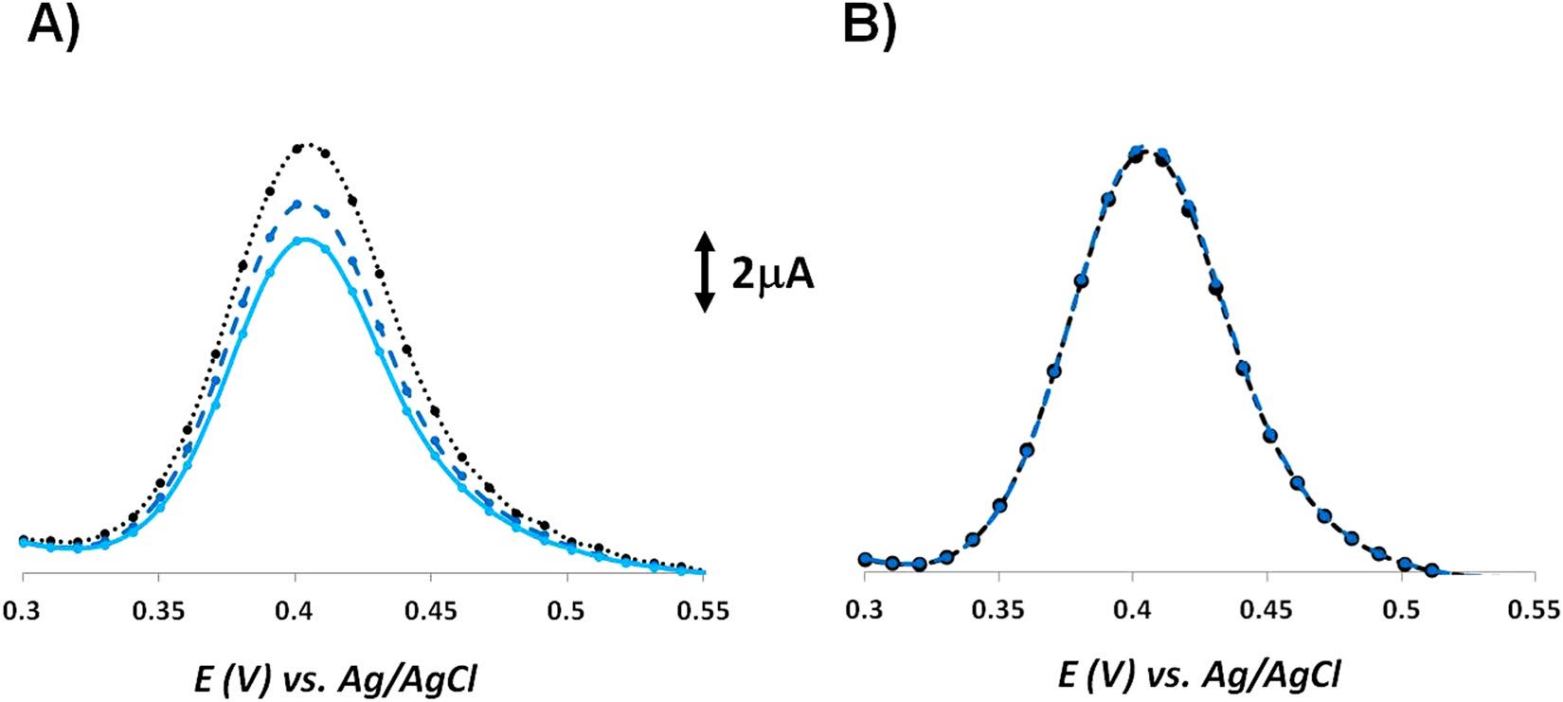
Successive differential pulse voltammograms of 0.1 mg/ml 8-OHdG in PBS pH 7.4 recorded (**A**) without any application of conditioning potential and (**B**) with a conditioning potential of +0.20 V applied before each measurement.

Here, the oxidation of 8-OHdG in pH 7.4 PBS was found to be around +0.41 V. Initially, the effect of performing a pre-conditioning step at a specific potential on the DPV electrochemical performance was investigated (Fig. 4). Our data showed that applying a conditioning potential of +0.20 V for pre-concentrating of 8-OHdG greatly improved the stability and reproducibility of the DPV measurement. Therefore, the subsequent DPV measurements were conducted over a positive potential range, after applying a fixed step potential for the pre-concentration of 8-OHdG on the surface of the modified electrodes.

Afterwards, the influence of the potential and accumulation time during the pre-concentration step on the electrochemical response of 8-OHdG was also investigated, as shown in Figure S2. Two different potentials were tested and the highest current signal was achieved at +0.20 V so, this potential value was chosen for further studies. Concerning the accumulation time, it was observed that the oxidation current increased from 180 s to 300 s and then it decreased for 500 s. Hence, the optimal time pre-concentration for the determination of 8-OHdG by DPV analysis was chosen to be 300 s.

Nowadays, one of the most popular electrochemical transducer in biosensor field is carbon-based surface (graphite, glassy carbon (GC), carbon paste). Due to their great stability and suitable electronic properties, these nanostructured materials have been widely applied, nevertheless, appropriate cleaning methodologies are still needed in order to enhance the material response. Herein, we have studied the effect of different kinds of washing solutions, such as, ethanol, buffers and acids, on the electrochemical response of our target molecule (data not shown). We have concluded that the best performance was achieved after applying voltammetric sweeps on PBS at a pH 7.4 (Figure S3A). More interestingly, by applying the same cleaning protocol after each DPV measurement (Figure S3B), we were able to completely regenerate the sensor by returning the current to its original level, enabling multiple use of the same electrode.

We have tested the performance of the DPV signal for different pH environments (Figure S4) and, as expected, we have found that the electrochemical oxidation of 8-OHdG is pH dependent. Our data seems to be in agreement with previous studies in which the anodic peak potential shifted towards negative values with the increase of the supporting electrolyte pH from 3.0 to 9.0, indicating that the electrochemical process of 8-OHdG is associated with a proton-transfer process^24^. As shown here, the tendency seems to be that the oxidation peak current decreases with the increasing of pH (Tris pH 9.5), has it was observed previously for guanine and adenine oxidation^21^. Although the maximum value of the current intensity was obtained for PBS when the pH was 6.0, it was quite similar when compared with the pH of 7.4. Thus, the physiological pH was selected and used in further studies.

### Analytical applications

#### Calibration curve

**Figure 5:**
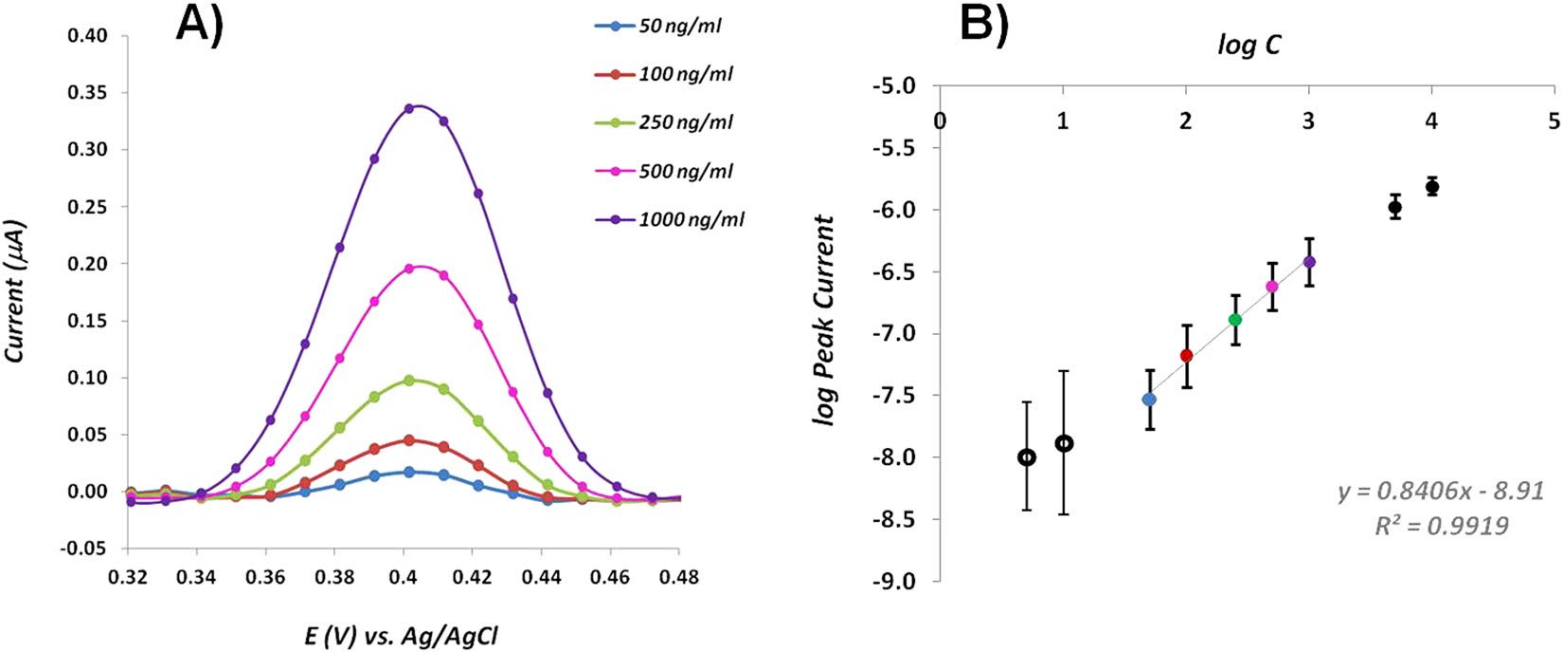
(**A**) Differential pulse voltammograms for different concentrations of 8-OHdG prepared in PBS pH 7.4 and (**B**) calibration plot of the concentration of 8-OHdG.

Figure 5A shows the corresponding dependence of the oxidation peak current on the concentration of 8-OHdG. As it can be seen, with the increase of concentration, the DPV signaling was also increased. Figure 5B displays the calibration graph for 8-OHdG showing a linear relationship between the log peak intensity and the log concentration of 8-OHdG over the range 50–1000 ng/ml. The calibration plot showed excellent linearity (r^2^ = 0.9919) and the limit of detection (*LOD*) was found to be 14.4 ng/ml, calculated by the intersection of two straight lines, between the linear response range and the lower concentration range. The reproducibility of the modified-electrodes for quantifying 8-OHdG was investigated over the entire linear range and data showed that the relative standard deviation (RSD) was less than 3.5%, for five independent experiments.

Although the *LOD* of our paper-based sensor might not fully satisfy the cut-off of all the biological samples, it has a relatively wide linear range and a good sensitivity compared to other works. Moreover, despite the fact that there are some known methodologies for 8-OHdG assessment down to picoMolar^17^, various clinical studies have reported that the levels of serum 8-OHdG in healthy subjects can suffer variations between 3 and 160 ng/ml^12,54–57^. Thus, our electrochemical label-free sensor is a valuable tool in providing quick and low-cost information concerning the level of OS at DNA context.

#### Selectivity

The selectivity of the proposed biosensor is crucial to grant its successful application. Meanwhile, one of the main limitations of most conventional methodologies involving biological samples is the need to include pre-treatment steps in order to minimize possible matrix interference effects. Herein, the target is to allow a direct sample analysis, with only dilution, if necessary.

**Figure 6:**
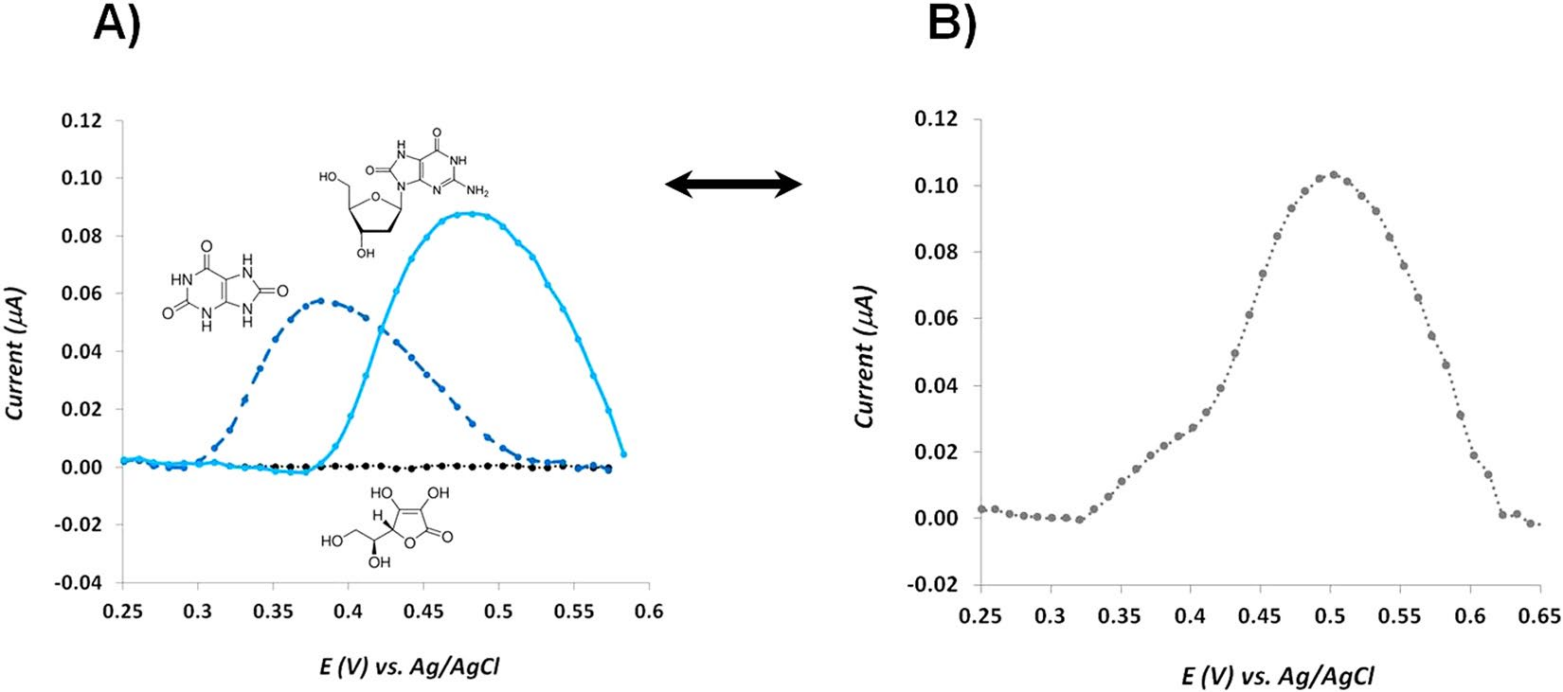
(**A**) DPV recordings for individual solutions with concentrations of 0.1 mM of 8-OHdG, ascorbic acid and uric acid in PBS at pH of 7.4; (**B**) DPV recording of a mixture with all of the 3 compounds, in the same concentrations.

Uric acid and ascorbic acid were chosen as main interfering species in the determination of 8-OHdG, due to their similar structure and high abundance in biological samples, respectively. Both molecules are also electro-active and, very often, they are oxidized almost at the same potential value, resulting in the overlap of voltammetric signaling. In order to test the selectivity behaviour of our sensor, we have investigated the oxidation peak potential of both target-molecule and interfering species, individually (Fig. 6A) and in an equimolar mixture (Fig. 6B). Our data showed that over the tested potential range, ascorbic acid did not exhibit any redox peak that could affect the analysis (Fig. 6A, black line). On the other hand, an oxidation peak was observed for the uric acid molecule (Fig. 6A, dashed dark blue line), but it occurs at a lower potential value compared with the oxidation potential of 8-OHdG (Fig. 6A, light blue line). Meanwhile, both molecules were analysed at the same concentration level (0.1 mM), however 8-OHdG presented an oxidation peak with higher current than uric acid. The differential pulse voltammogram obtained from the equimolar mixture of the three molecules showed that the oxidation potential peak of 8-OHdG in the mixture was shifted to values above +0.5 V may be due to the presence of high concentration of uric acid. Concerning the peak intensity current, the same value was obtained in comparison with the individual solution. In addition, the oxidation peak of uric acid was not visible but instead a small shoulder appeared at lower potentials, enabling a clear separation between the oxidation process of 8-OHdG and uric acid.

#### Serum samples

In order to investigate the applicability of our paper-modified electrode for assessment of 8-OHdG levels in POC, we performed some electrochemical studies using real serum samples. Biological fluids, such as, urine, blood and serum are complex matrices, composed by high levels of small biomolecules, some of which are electrochemically active and thereby act as possible interfering species. Thus, to optimize some experimental conditions, we have tested the dependence of the oxidation peak potential of both the target-molecule and possible interferent species for different electrolytes and pH values, aiming for a good voltammetric separation of the compounds. Figure S6 shows the differential pulse voltammograms obtained for 8-OHdG spiked serum samples 1:10 diluted in different buffers at different pH environments (basic, neutral and acid). Although it is not displayed here, all the tested diluted serum samples presented a broad DPV signal between +0.35 V and +0.45 V due to the presence of other interferent species holding electroactivity characteristics, such as, ascorbic acid and uric acid, among others. Our data showed that by doping these serum samples with a known concentration of 8-OHdG we were able to obtain a second DPV peak assigned to the oxidation of our target-molecule, with the exception of the buffer Tris pH 9.1 that presented no extra peak (Figure S6A). This outcome is in agreement with our previous pH study showing that the oxidation peak current is negatively affected by the increasing of pH. For the other tested pH values (Figure S6B and S6C), the best enhancement in the oxidation current of 8-OHdG was achieved for the sample diluted in PBS pH 7.4. The obtained results are in good agreement with other similar studies that showed that the peak current of 8-oxoguanine, in the presence of interferents, was maximum in the pH range 6–8^58^. In sum, PBS at 7.4 pH was the optimal choice for 8-OHdG electrochemical detection since the peak current is higher and sufficiently good separation between peaks was reached.

**Figure 7:**
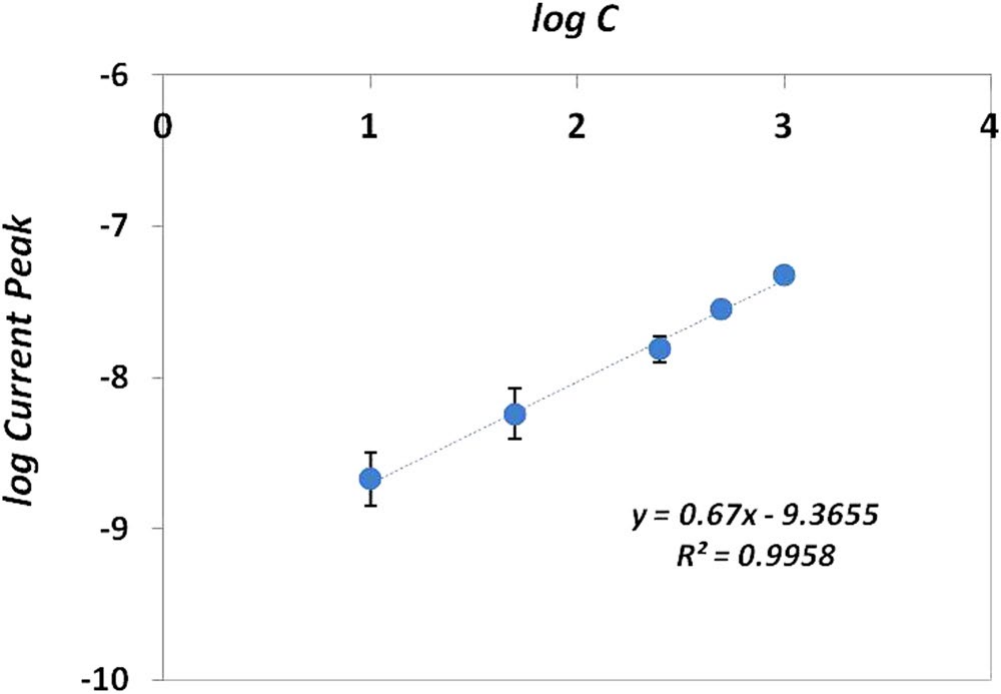
Calibration curve of the concentration of 8-OHdG in diluted serum samples.

Meanwhile, the application of the proposed sensor in the quantification of 8-OHdG was tested in serum samples by using a spiking approach. All samples were previously diluted 1:10 in PBS pH 7.4. Three independent measurements were performed for each concentration. Figure 7 presents the calibration graph for 8-OHdG concentration obtained from the DPV response. A linear regression equation (*log current (A) = 0.67 log concentration (ng/ml)−9.3655*) was achieved over the concentration range 10–1000 ng/ml with a correlation coefficient of 0.9958. Moreover, the reproducibility of the modified-electrodes was confirmed and RSD was less than 2.5%.

Although other works have been reported with better sensitivity (see Table 1), they have used more complex and expensive protocols in comparison with our paper-based sensor. For instance, some interesting studies related to the modification of GCE surface with materials that effectively increased their surface area enabled the detection of 8-OHdG molecule at very low levels (0.1–0.2 ng/ml) but in both cases the immobilization of antibodies was performed^14,59^. One of the main advantages of our 3-electrode system assembled on paper substrate is their ability to work with small sample volumes. Moreover, there are very few papers concerning the application of rapid and cost-effective biosensing devices for real serum samples. Thus, the analysis of 8-OHdG in serum samples was successfully achieved in less time and with low cost input, allowing the detection of OS biomarkers in biological samples.

## Conclusions

We have investigated the electrochemical performance of 8-OHdG biomarker at the surface of paper-modified electrodes for *in-situ* detection purposes. Different carbon-based nanomaterials were tested for coating the paper support and it was found that the presence of the conducting polymer PEDOT could effectively enhance the oxidation peak current of 8-OHdG. In parallel, several experimental conditions, such as, scan-rate, potential of pre-accumulation, accumulation time, supporting electrolyte and pH have been carefully optimized and the electrochemical response of the designed sensor was investigated by means of DPV. Overall, the combination of electrical properties of PEDOT with the electrochemical sensing of 8-OHdG enhanced the electro-catalytic activity of the working electrode, which resulted in favourable analytical features, such as, high reproducibility and selectivity, with low cost resources.

One main advantage of this biosensor is easy and quick way to regenerate it, simply by performing voltammetric cycles in buffer solution, enabling continuous real-time detection of several samples. The developed electrochemical paper-based sensor showed high sensitivity towards 8-OHdG over the concentration range 50–1000 ng/ml, enabling low detection limit. Thus, the proposed electrochemical sensor holds high selectivity, reproducibility and stability, which constitutes a promising low-cost approach to be implemented as an easy-to-use protocol for sensitive detection of 8-OHdG in biological samples.

## Methods

### Reagents

All reagents were of analytical grade and used without further purification. Phosphate buffered saline (PBS, 0.01 M, pH 7.4), TRIS (hydroxymethyl)aminomethane (TRIS, 0.01 M, pH 9.1 and 9.5) and acetate buffer (1 mM, pH 5.1) were used as buffer solutions and prepared with ultrapure water Mili-Q laboratory grade. The pH values were measured with a pH meter (Crison Instruments, GLP 21 model). Graphite powder (fine extra pure, particle size < 50 μm) was purchased from Merck and used as received. Poly(3,4-ethylenedioxythiophene) (PEDOT) nanoparticles dispersion in H_2_O, 8-hydroxy-2-deoxyguanosine (8-OHdG, 98%), uric acid (>99% crystalline), sulphuric acid (H_2_SO_4_, 95–97%), multi-walled carbon nanotube (MWCNT) and multi-walled carbon nanotube, carboxylic acid functionalized (MWCNT-COOH) were obtained from Sigma Aldrich; poly(vinyl chloride) carboxylated (PVC-COOH) from Fluka; *N,N*-dimethylformamide (DMF) from Analar Normapur and ascorbic acid from Riedel-de-Haen. All measurements were carried out at ambient temperature.

### Apparatus

Electrochemical measurements were performed by using a three-electrode system composed by a carbon-ink coated paper with an electrode area of 20 mm^2^ as the working electrode, a Pt wire as the counter electrode and an Ag/AgCl (KCl 3.0 M) wire as the reference electrode. All the electrochemical measurements including cyclic voltammetry (CV) and differential pulse voltammetry (DPV) experiments were conducted with a potentiostat/galvanostat from Metrohm Autolab and a PGSTAT302N with a FRA module, controlled by ANOVA software.

### Fabrication and characterization of the paper-based sensor

The working electrode was constituted by small parts of cellulose paper (5 × 2 mm) hand-coated with a conductive carbon-based ink. The protocol for the preparation of this conductive carbon-based surface is described elsewhere^49^. Briefly, graphite powder was doped with PVC-COOH and dispersed in DMF with magnetic stirring at room temperature. In addition, a small amount of different nanostructured materials were added (separately) to the previous mixture and left stirring for several hours before using. Specifically, we have tested PEDOT nanoparticles dispersed in water, MWCNT and MWCNT-COOH powders. The sensor was prepared by drop-coating the surface of the hydrophobic paper with the conductive carbon-based ink. Afterwards, the electrode area was precisely delimited by using paraffin wax.

Carbon-coated paper was characterized by means of RAMAN spectroscopy. The Raman spectral analysis was carried out using a Thermo Scientific DXR Raman microscope system with a 100 mW 532 nm excitation laser (operational conditions: 20 min of photobleach and 5 min of collect time). Data analysis was performed with OMNIC software.

### Electrochemical assays

Initially, the working electrode was electrochemically cleaned by performing voltammetric sweeps between −0.2 V and +1.5 V in PBS pH 7.4, until a stable voltammogram was obtained (more or less 50 cycles). Before use, sensors were dried and stored at room temperature.

The electrochemical response of 8-OHdG in PBS solution at pH 7.4 was investigated by performing CV measurements over the potential range of +0.1–+0.8 V, in order to find the oxidation potential. During the electrochemical measurements the three-electrode system was always submersed into 1 ml of sample volume. Afterwards, differential pulse voltammograms were recorded after pre-conditioning the working electrode in 8-OHdG solution at a specific potential. The DPV experimental conditions used were a potential range between +0.2 V and +0.7 V, pulse amplitude of 25 mV, pulse width of 50 ms, scan-rate of 100 mVs^−1^ and an equilibration time of 5 s. In order to regenerate the sensors, a cleaning protocol was performed to remove the analyte adsorbents by immersion of the used sensors in 0.01 M PBS pH 7.4, followed by (5) successive CV scannings over the potential range 0 –+0.7 V at a scan-rate of 50 mVs^−1^.

Calibration curves were performed by DPV analysis for 8-OHdG in the range 50–1000 ng/ml in PBS solution at pH 7.4 and all the logarithmic scales were calculated from ng/ml values. Selectivity studies were carried out by competitive assays between 8-OHdG (0.1 mM) and each interfering specie, with similar concentration. Here, uric acid and ascorbic acid were selected as interfering molecules due to the fact that they may co-exist with 8-OHdG in biological fluids, holding also electro-active properties. Additionally, different buffer solutions were prepared at different pH environments, such as, PBS at neutral pH (pH 7.4, 10 mM), Tris buffer at basic pH (pH 9.1, 2 mM) and Acetate buffer at acidic pH (pH 5.1, 1 mM). The performance of the sensor was tested directly in Foetal Bovine Serum doped with 8-OHdG in the concentration range 20–1000 ng/ml. The serum was previously 1:10 diluted in PBS buffer.

## Electronic supplementary material

Supplementary Information
