# Supplementary material for: Paper-Based Sensing Device for Electrochemical Detection of Oxidative Stress Biomarker 8-Hydroxy-2′-deoxyguanosine (8-OHdG) in Point-of-Care
Source: Sci Rep. 2017 Nov 6;7:14558. doi: 10.1038/s41598-017-14878-9 (PMC5673927; doi:10.1038/s41598-017-14878-9)
Supplement: Supplementary file 1 — Supplementary Information [file 41598_2017_14878_MOESM1_ESM.pdf]

Paper-Based Sensing Device for Electrochemical Detection of Oxidative Stress Biomarker 8-Hydroxy-2'-deoxyguanosine (8-OHdG) in Point-of-Care

Gabriela V. Martins[1,2], Ana P. Tavares[1], Elvira Fortunato[2], M. Goreti F. Sales[1,*]

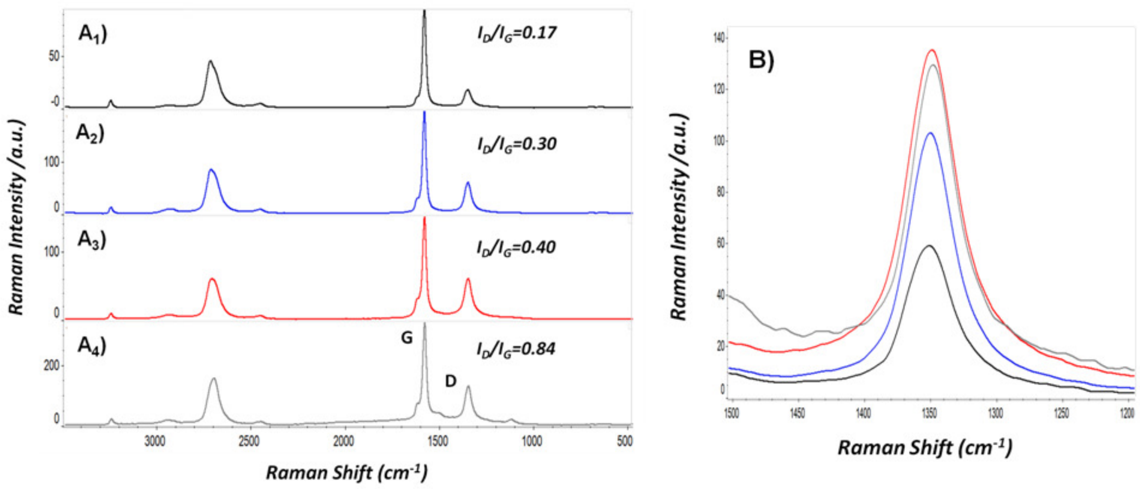

SUPPLEMENTARY FIGURE S1: RAMAN spectra of the different graphite-based electrodes prepared after the incorporation of nanomaterials dispersed in the graphite ink, such as, A1) PEDOT nanoparticles, A2) Graphite, A3) CNTMW and A4) COOH-CNTMW, with the calculated I_D/I_G ratios and B) RAMAN spectra with the magnification of the D (Disorder) band, in full-scale mode.

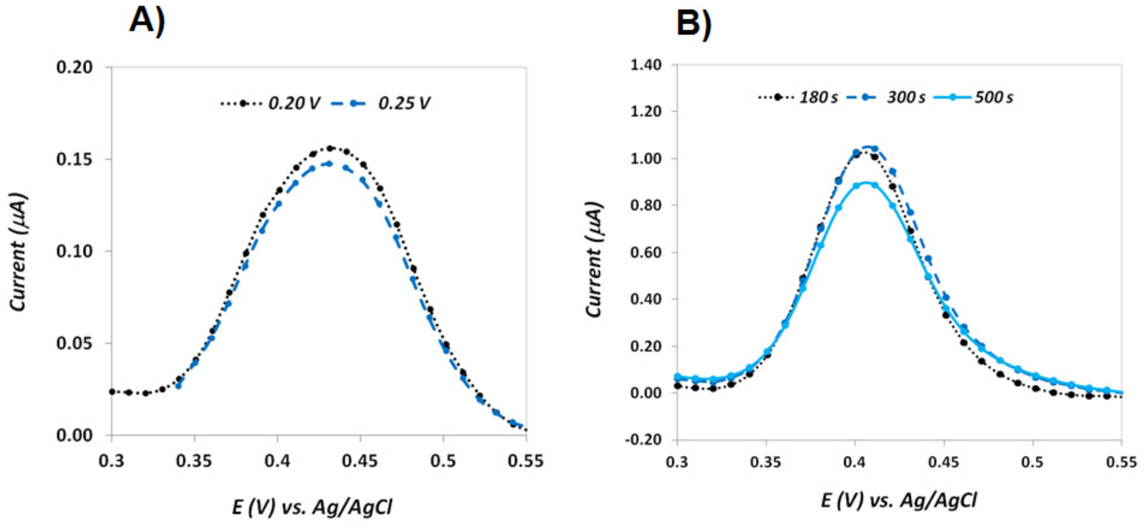

SUPPLEMENTARY FIGURE S2: Dependence of the sensor response on the (A) pre-accumulation potential and (B) time of accumulation during 8-OHdG oxidation in PBS pH 7.4.

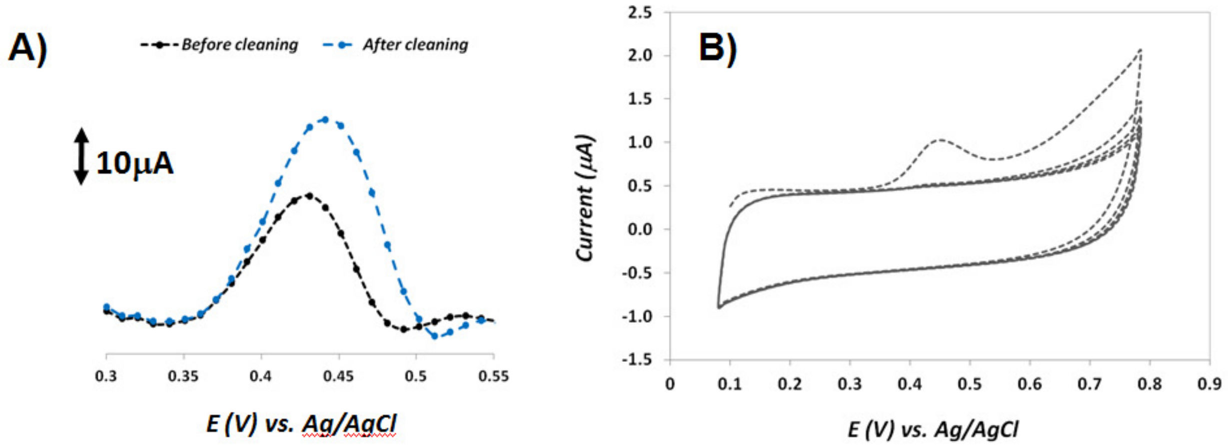

SUPPLEMENTARY FIGURE S3: (A) Cleaning effect (after CV in PBS pH 7.4) on the 8-OHdG detection by DPV signal and (B) sensor regeneration after voltammetric cycles performed in PBS pH 7.4.

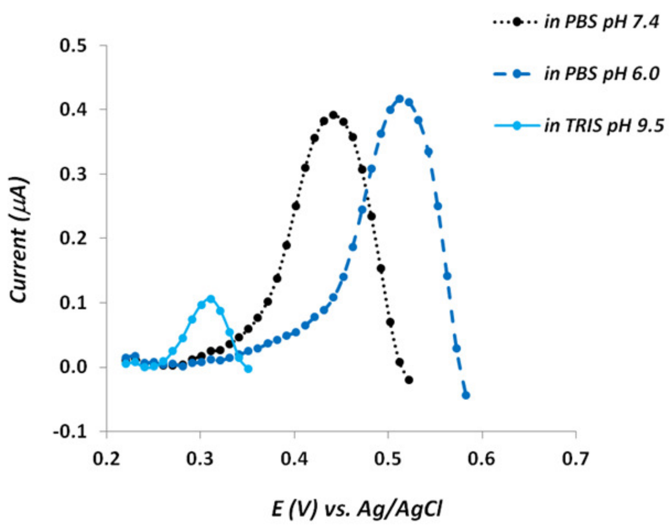

SUPPLEMENTARY FIGURE S4: Differential pulse voltammograms recorded for 8-OHdG solutions prepared in different buffer solutions, with different pH values.

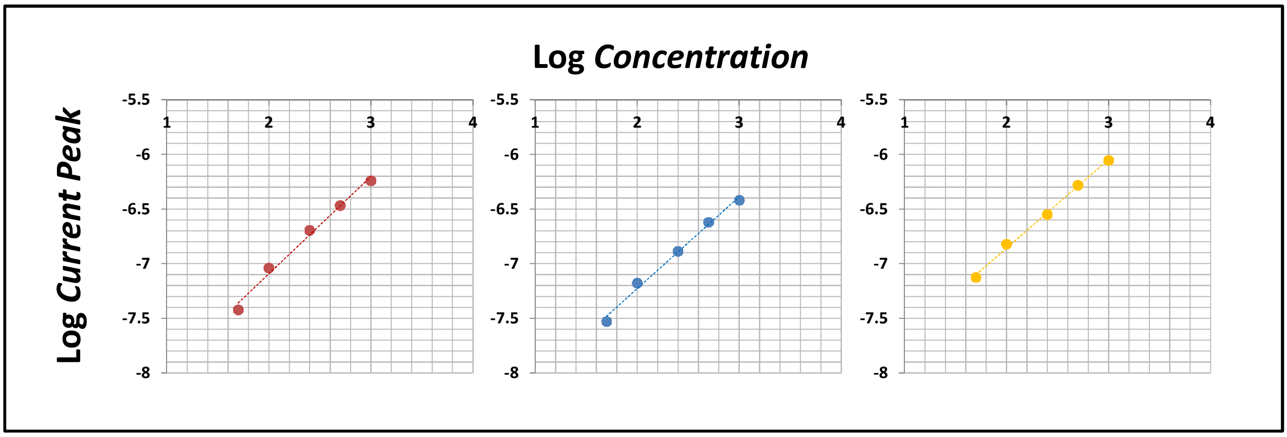

SUPPLEMENTARY FIGURE S5: Three independent calibration plots of the concentration of 8-OHdG prepared in PBS pH 7.4.

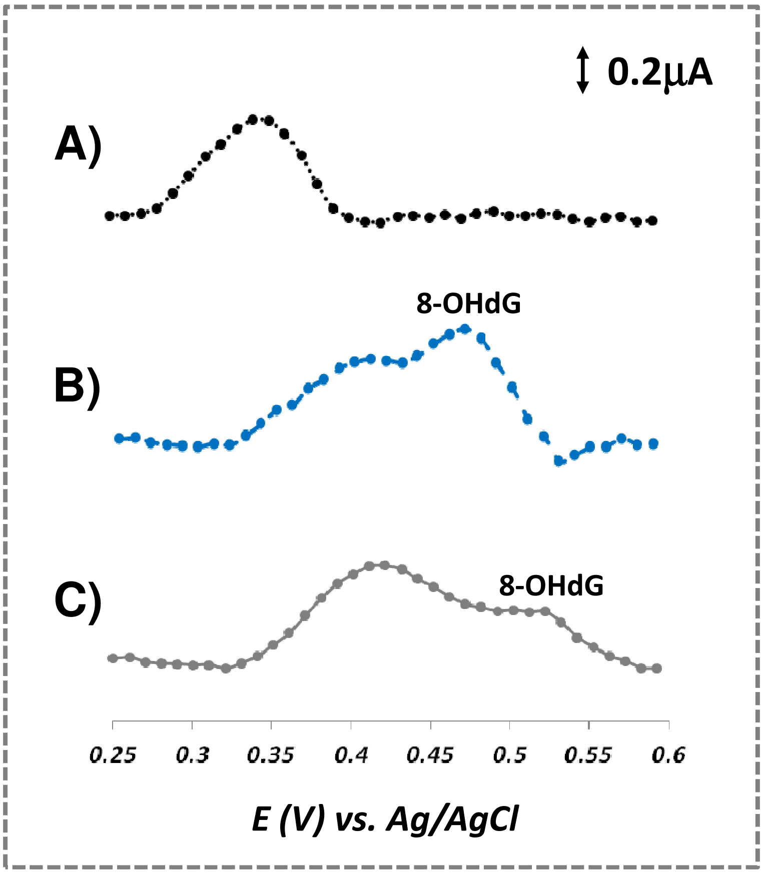

SUPPLEMENTARY FIGURE S6: Differential pulse voltammograms for serum samples diluted 1:10 in different buffers, such as, (A) Tris pH 9.1, (B) PBS pH 7.4 and (C) Acetate pH 5.1, doped with 1 ug/ml of 8-OHdG.
